# What are the barriers preventing the screening and management of neonatal hypoglycaemia in low-resource settings, and how can they be overcome?

**DOI:** 10.1186/s40748-023-00162-4

**Published:** 2023-06-01

**Authors:** Lauren M Irvine, Deborah L Harris

**Affiliations:** 1 School of Nursing, Midwifery, and Health Practice, Faculty of Health, Victoria University of Wellington – Te Herenga Waka, Deborah Harris Level 7, Clinical Services Block, Wellington Regional Hospital, Newtown, Wellington, 6021 New Zealand; 2 Liggins Institute, University of Auckland, Auckland, New Zealand

**Keywords:** Health equity, Newborn, Blood glucose monitoring, Dextrose gel, Developing nations, Neonatal care

## Abstract

Over 25 years ago, the World Health Organization (WHO) acknowledged the importance of effective prevention, detection and treatment of neonatal hypoglycaemia, and declared it to be a global priority. Neonatal hypoglycaemia is common, linked to poor neurosensory outcomes and, if untreated, can cause seizures and death. Neonatal mortality in low and lower-middle income countries constitutes an estimated 89% of overall neonatal deaths. Factors contributing to high mortality rates include malnutrition, infectious diseases, poor maternal wellbeing and resource constraints on both equipment and staff, leading to delayed diagnosis and treatment. The incidence of neonatal hypoglycaemia in low and lower-middle income countries remains unclear, as data are not collected.

Data from high-resource settings shows that half of all at-risk babies will develop hypoglycaemia, using accepted clinical thresholds for treatment. Most at-risk babies are screened and treated, with treatment aiming to increase blood glucose concentration and, therefore, available cerebral fuel. The introduction of buccal dextrose gel as a first-line treatment for neonatal hypoglycaemia has changed the care of millions of babies and families in high-resource settings. Dextrose gel has now also been shown to prevent neonatal hypoglycaemia.

In low and lower-middle income countries, there are considerable barriers to resources which prevent access to reliable blood glucose screening, diagnosis, and treatment, leading to inequitable health outcomes when compared with developed countries. Babies born in low-resource settings do not have access to basic health care and are more likely to suffer from unrecognised neonatal hypoglycaemia, which contributes to the burden of neurosensory delay and death.

## Introduction

More than 25 years ago, the World Health Organization (WHO) attested that preventing neonatal hypoglycaemia, and developing methods of screening and treating the condition without harming the establishment of breastfeeding to be a global priority [1]. Neonatal hypoglycaemia is the most common metabolic condition in newborns [2], causing significant long-term disability, seizures and death which is largely preventable [3–7]. Since this statement from the WHO, there has been considerable research in high and upper-middle income countries (HUMIC) advancing knowledge surrounding the prevention, treatment and management of neonatal hypoglycaemia [8–12]. This has changed clinical practice for millions of babies and families in such countries. However, in low and lower-middle income countries (LLMIC), evidence related to the incidence, prevention, treatment and long-term outcomes of neonatal hypoglycaemia remain largely unclear and data are scarce. This directly impacts two-thirds of the estimated 135 million babies born each year, who are born in LLMIC [13].

The neurosensory impairments associated with neonatal hypoglycaemia in low resource settings are often unrecognised and unmanaged. There is evidence showing symptomatic, severe or prolonged episodes of hypoglycaemia can cause neurological injury leading to neurological sequelae including infantile spasms, acute seizures, drug resistant epilepsy [14], brain injury and death [15, 16]. Yet, neurosensory impairment related to transitional hypoglycaemia in HUMIC has caused considerable controversy [5, 17], and remains unclear. However, recent findings from the Hypoglycaemia Prevention with Oral Dextrose (hPOD) follow-up study, which sought to determine neurosensory outcomes in children who, as babies, received either 40% dextrose or placebo gel in the first 48 h after birth, showed those who became hypoglycaemic (< 47 mg/dl, < 2.6 mmol/l) were more likely to have neurosensory impairment [18]. Furthermore, children who had experienced more severe episodes were at an increased risk of impairment. This suggests that children in HUMIC settings, receiving high quality healthcare to maintain normoglycaemia, are at risk of neurological harm when cerebral fuels are compromised even for short periods.

The physical and economic disparities in low-resource settings prevent access to reliable prophylactic strategies [19], blood glucose screening [20–25], diagnosis [20–25] and treatment [20, 21, 24–27]. Therefore, unlike babies born in HUMIC, babies in low-resource settings are more likely to suffer from unrecognised neonatal hypoglycaemia and remain untreated, leading to poor health outcomes and death [6]. As there is little consensus on the definition of low-resource settings, this discussion identifies LLMIC using the world bank classification [28], and uses the terms LLMIC and low-resource settings interchangeably.

### Risk factors and incidences of neonatal hypoglycaemia

Babies considered at-risk of developing hypoglycaemia include those unwell, infants of diabetic mothers, those born small (SGA; weighing < 10th centile and/or < 2.5 kg) or large for gestational age (LGA; >90th centile and/or > 4.5 kg) [29], those born prematurely (< 37 weeks’ gestation), or those not feeding well [10]. In HUMIC settings, neonatal hypoglycaemia is common, with half of the at-risk babies likely to be diagnosed within the first 48 h after birth [10]. Screening for hypoglycaemia is recommended for at-risk babies using capillary heel-prick lancing to sample blood for glucose concentration analysis, along with close monitoring for clinical signs.

While evidence reporting the incidence of neonatal hypoglycaemia in LLMIC are few, it is likely to be higher than in HUMIC due to the high prevalence of risk factors which predispose babies to hypoglycaemia. It is also possible that factors which influence the risk of neonatal hypoglycaemia vary within and between LLMIC countries. For example, Pacific Islanders as an ethnic group have high prevalence rates of gestational diabetes mellitus (GDM) and macrosomia [30]. While, in Pakistan, high rates of preterm births, intrauterine growth restriction, infections, sepsis, and perinatal asphyxia are reported [31]. Programs including the Early Essential Newborn Care (EENC) developed by WHO and UNICEF which seek to improve routine neonatal care in LLMICs are being successfully implemented, and report lifesaving improvements in nine priority action countries with high neonatal mortality rates [32]. It is likely that these programs are contributing to reducing the burden of dysglycaemia.

### Screening and diagnosis of neonatal hypoglycaemia

The greatest barrier to effective screening and diagnosis of neonatal hypoglycaemia in low-resource settings is the scarcity of healthcare resources, equipment, and staffing. Investigators seeking to assess the quality of care for hospitalised babies treated for infections in Bangladesh, Nepal, and Tanzania showed an absence of basic bedside diagnostics for the screening of hypoglycaemia [25]. Despite these babies being identified at-risk for hypoglycaemia, evidence of blood glucose screening ranged from only 1–51% across the five hospitals assessed. Additionally, of the LBW babies (n = 1015), 905 (89%) were admitted with sepsis, surprisingly the diagnosis of neonatal hypoglycaemia was reported 0–9% across the five hospitals [25]. This can be compared to hypoglycaemia diagnoses of 36–72% in babies with infections in other low-resource settings [33–35] providing evidence of countless unwell babies with unrecognised and untreated hypoglycaemia.

Similarly, in Kenya, the provision of basic diagnostics including blood glucose screening is often unavailable [22] and, in Nepal, only high risk babies such as those LBW and post term are reportedly screened for hypoglycaemia at the cot side due to limited resources [23]. A four-year neonatal mortality audit in Gambia found that supplies of glucometer strips were frequently exhausted, and < 70% of babies were screened for hypoglycaemia on admission [24]. Together, these studies highlight the systemic barriers in low-resource settings and the necessity to prioritise investments into basic diagnostics such as reliable blood glucose analysers. Targeted aid in these low-resource settings is urgently needed to address the inequity in the care provided, despite such provisions being included in the United Nation’s Sustainable Development Goal (SDG) 3 – “ensure healthy lives and promote well-being for all, at all ages”. Concerningly, a recent UNICEF review reports many countries are not meeting targets [36].

Efforts to develop an ideal blood glucose analyser for LLMIC settings, one that is non-invasive, reliable, portable and inexpensive, have been largely unsuccessful [37]. However, recent advances on a device utilising light-based senses to measure blood glucose concentrations through skin contact are encouraging [38]. Gluco-Light devices involve a one-off expense estimated to be $318 USD replacing the necessity for disposable supplies of frequently unavailable glucose strips. The Gluco-Light provides instantaneous results, ensuring timely treatment, and determines response to feeding and real-time titration of medications, if required. Further, the device is simple to use, and requires limited training. Other benefits include decreasing the repetitive experiences of heel-prick glucose lances which can be painful and have associated risks [39], reducing the risk of infection, and decreasing parental anxiety [40]. The accuracy and reliability of the Gluco-Light in neonatal populations is currently being evaluated [41]. Implementation of non-invasive point-of-care analysers into routine neonatal care in both high and low-resource settings is expected to significantly change current clinical practice.

### Clinical practice guidelines and diagnostic parameters

In HUMIC, clinical guidelines are used, supporting an evidenced-based approach to the management of neonatal hypoglycaemia [42–45]. In low-resource settings, clinical guidelines may not be practicable due to financial, resource and staffing constraints. A report from the WHO shows guidelines in low-resource settings are often unsuccessfully implemented due to insufficient support from management, healthcare provider denial and disagreement with guidelines, as well as incompatibility to local conditions and resources [46]. In Kenya, despite staff being aware of current evidence-based practices for neonatal hypoglycaemia, the lack of basic resources and equipment prevents guideline adherence [22]. Therefore, while evidence-based guidelines facilitate improved clinical decision making and standardised patient care [47], if they are to be useful in low-resource settings, it is essential they are compatible with available resources, and relevant cultural norms.

There remains considerable variation regarding the threshold for diagnoses of neonatal hypoglycaemia in LLMIC (Table 1), which is also evidenced in guidelines across high-income countries [43, 45, 48]. Further, when blood glucose screening is available in LLMIC it is frequently measured using unreliable point-of-care analysers (Table 1), which both under- and over-estimate blood glucose concentration. In a rural hospital in Kenya, the absence of adequate diagnostic equipment was found to result in increased morbidity and mortality in children and neonates with hypoglycaemia [21]. Therefore, presumptive treatment is recommended for children and neonates with severe illness in the absence of diagnostic equipment. Although this type of routine presumptive treatment is controversial, the risks associated with untreated hypoglycaemia are substantial when compared to the relative risks of treating suspected hypoglycaemia with non-invasive, safe and effective methods such as buccal 40% dextrose gel.

**Table 1:** The differing diagnoses of neonatal hypoglycemia, and blood glucose analyser used in low resource settings

Study	Country	Neonates (n)	Diagnosis	Analyser	Risk factors
Bora et al., 2020 [26]	India	80	< 40 mg/dL (< 2.2mmol/L)	POC electrochemical, glucose oxidase	SGA
Ellis et al., 1996 [23]	Nepal	94	< 36 mg/dL (< 2.0 mmol/L)	POC electrochemical, reflectance meter and glucose oxidase	-
Gupta et al., 2022 [27]	India	629	< 45 mg/dL (< 2.2mmol/L) in neonates > 24 h < 25 mg/dL (< 1.4mmol/L) 0 to 4 h	POC electrochemical	SGA, LGA, intrauterine growth-restriction, infants of diabetic mothers, late preterm (> 35 weeks)
Ibrahim et al., 2021 [31]	Pakistan	120	< 40 mg/dL (< 2.2mmol/L)	Laboratory	Preterm neonates (Mean gestational age was 32.3 + 6.37 weeks)
Mukunya et al., 2020 [62]	Uganda	1416	< 47 mg/dL (< 2.6mmol/L)	POC electrochemical	Delayed establishment of breastfeeding, < 3 days of age
Okomo et al., 2015 [24]	Gambia	4944	< 47 mg/dL (< 2.6mmol/L)	POC (analyser not specified)	-
Osier et al., 2003 [21]	Kenya	280	< 40 mg/dL (< 2.2mmol/L)	Glucose oxidase	Unable to establish breastfeeding, SGA
Pal et al., 2000 [61]	Nepal	578	Mild < 47 mg/dL (< 2.6mmol/L) Moderate < 36 mg/dL (< 2.0 mmol/L)	Glucose oxidase	Postmaturity, SGA, delayed establishment of breastfeeding, small head size, haemoglobin > 210 g/l, raised maternal thyroid stimulating hormone
Sasidharan et al., 2004 [19]	India	604	< 40 mg/dL (< 2.2mmol/L)	Glucose oxidase	LBW, preterm, maternal diabetes, maternal oligohydramnios, pre-eclampsia, birth asphyxia, cold stress, hypothermia, delayed establishment of breastfeeding

LBW: low birth weight; POC: point of care; SGA: small for gestational age

## Prevention of neonatal hypoglycaemia

Strategies which may reduce the incidence of hypoglycaemia in both at-risk and healthy babies include early skin-to-skin contact [49, 50], keeping babies warm and dry, and establishing early breastfeeding [51]. Additional strategies have been investigated. Authors from India who sought to reduce neonatal hypoglycaemia randomised small or large for gestational age babies to either feeding with infant formula or infant formula with additional powdered sugar, showing those who received the additional powdered sugar were less likely to become hypoglycaemic [52, 53]. Further, it has also been proposed, that supplementary feeding will reduce the risk of hypoglycaemia [44]. However, both formula and supplementary feeding have been associated with negative implications on both the establishment and duration of breastfeeding [54, 55]. Breastfeeding is universally recommended [56, 57], yet, the relationship between breastfeeding and changes in blood glucose concentrations remains unclear and controversial [58, 59]. A recent report about the feeding patterns of healthy term newborns, shows breastfeeding for durations of > 30 min increases blood glucose concentrations in the first days following birth [60]. However, further investigation is warranted to affirm these feeding patterns as recommendations for newborns at risk of hypoglycaemia.

Resource constraints in LLMIC mean strategies that prevent babies from developing hypoglycaemia are unable to be practiced effectively. For example, breastfeeding is often delayed and there is inadequate prevention of hypothermia [19, 61, 62]. Many home births in LLMIC, do not have the support of healthcare professionals, and following birth, access to hospital care is difficult, requiring long distances to be travelled to the nearest hospital, which are frequently overcrowded. Therefore, mothers and babies are discharged early to accommodate those who are seen requiring critical care, resulting in a lack of support to establish breastfeeding [14, 63]. This further increases the risks of neonatal hypoglycaemia. A focus on postnatal education and a comprehensive discharge plan for parents would likely decrease incidences of neonatal hypoglycaemia and provide improved health outcomes for mother and baby.

### Dextrose gel as a prophylaxis

We are not aware of any clinical trials investigating the use of dextrose gel as prophylaxis in LLMIC settings. In high income settings, prophylaxis with buccal dextrose gel in late preterm and term babies in the first 48 h after birth has been shown to reduce the incidence of hypoglycaemia [11]. Authors reported that those babies who were randomised to dextrose gel were less likely to become hypoglycaemic (blood glucose concentration (< 47 dl/ml, (< 2.6 mmol/L)) [dextrose 399/1,070, (37%) v. placebo gel 448/1063, (42%); aRR 0.88; 95%CI, 0.80, 0.98; p = 0.02)]. Babies who received the dextrose gel were not at increased risk of recurrent or more severe episodes of low glucose concentrations when compared with babies who received placebo gel, suggesting that prophylaxis with dextrose gel may support glucose stability soon after birth. Importantly, prophylaxis with dextrose gel, did not harm the establishment of breastfeeding, or breastfeeding up to six weeks after discharge from hospital. In fact, research instead demonstrates that dextrose gel supports early breastfeeding, a crucial aspect in the prevention of hypoglycaemia [64, 65].

### **Treatment for neonatal hypoglycaemia**

In the developed world, the most common treatment for neonatal hypoglycaemia is feeding, plus or minus oral dextrose gel. If the blood glucose concentration remains low, then admission to the newborn intensive care unit is normally required for treatment with intravenous dextrose. In LLMIC, equipment, staffing and resource shortages are barriers to providing IV dextrose due to the required specialised skills and equipment.

Establishing IV access in babies is often lifesaving, but difficult and has been shown to be frequently unfeasible in low-resource settings [20, 26, 66, 67]. A report from Gambia attributed incidences of hypoglycaemia in at-risk babies was in part due to the inability of staff being able to establish IV access [24]. Further, the hospital was poorly staffed, and those clinicians managing sick babies had insufficient training and limited knowledge, leading to omitted investigations and poor recognition of conditions such as hypoglycaemia. Therefore, the babies received less than ideal or ineffective treatment. In Kenya, presumptive treatment with intravenous or nasogastric dextrose is recommended for severely ill or malnourished neonates and children in the absence of diagnostic equipment [21].

A recent UNICEF report details that, in low-resource settings, syringe pumps are commonly unavailable, therefore intravenous fluids are delivered via slow push, burette or gravity-fed. This can lead to iatrogenic harm including inaccurate dosing and complications such as fluid overload [67]. These factors contribute to the 2.4 million neonatal deaths annually, half of which are caused by lack of access to simple interventions and quality care [68]. These findings suggest that an oral treatment such as dextrose gel, which is easy to administer, could be a useful alternative as first-line treatment for neonatal hypoglycaemia in LLMIC.

Ideally, low resource settings require an effective, easy to produce, low-cost oral treatment which supports breastfeeding while reducing the need for specialised and scarce resources. Across the developed world, since the publication of the Sugar Babies Study in 2013 [9], 40% dextrose gel and feeding have become first-line treatment for neonatal hypoglycaemia [4, 42, 44, 45, 69]. The Sugar Babies study showed that 40% dextrose gel (200 mg/kg) rubbed directly to the buccal mucosa, together with feeding, reverses neonatal hypoglycaemia, reduces maternal/neonatal separation and supports breastfeeding in late preterm and term babies within the first 48 h after birth. In HUMIC, if hypoglycaemia is severe or persistent, intravenous (IV) dextrose is normally administered in the neonatal intensive care unit [4, 42, 44, 45, 69]. A cost analysis of dextrose gel as an initial treatment determined it reduces hospital costs by an estimated $825 USD per episode treated [70] it is, therefore, considered a cost effective initial treatment.

### 40% dextrose gel in low resource settings

There is no evidence that 40% dextrose gel is used as prophylaxis or treatment for neonatal hypoglycaemia in the low-resource settings; perhaps because it is reported to be difficult to access and import [27]. The use of alternative fast-acting carbohydrate treatments has been reported. A randomised controlled trial in India sought to compare the efficacy of oral sucrose combined with expressed breastmilk, with 10% IV dextrose, in 80 premature (> 32 to < 36 weeks) SGA (> 1.2 to < 2.5 kg) hypoglycaemic babies (< 40 mg/dL, < 2.2mmol/L) [26]. The authors showed the oral treatment was possible, with no significant difference in the incidence of recurrent hypoglycaemia between these two treatment groups, demonstrating that oral sucrose could be effective in maintaining euglycemia in the context of low-resource settings. Similarly, a randomised trial comparing oral, sublingual and IV dextrose administration demonstrated the efficacy of sublingual sugar in resolving moderate hypoglycaemia(40-80 mg/dL or 2.2-4.4mmol/L) in children with malaria and respiratory tract infections [71]. Prompt and effective treatment is critical in this population, as hypoglycaemia is a life-threatening complication of infectious diseases such as malaria, causing coma and death [72].

While the use of such alternatives represents innovation and resourcefulness on the part of practitioners, there is an absence of robust clinical trials supporting their safety and efficacy in routine clinical practice. In contrast, oral treatment with dextrose gel has been shown to be safe soon after birth, and robust follow-up studies have reported no adverse clinical effects in children who received dextrose gel, as babies [8, 73, 74]. Additionally, dextrose gel is inexpensive, easy to administer, reduces the separation of mothers and babies [9], and supports the establishment of early breastfeeding [65]. Dextrose gel can be inexpensively prepared within hospital pharmacy services [75]. The gel is prepared through a combination of glucose thickened with a vehicle gel carboxymethylcellulose, with added citric acid as the preservative, creating a stable gel that lasts for 30 days. Thus, a potential way to overcome these access related barriers in hospitals with pharmacies is to educate and train staff on how to make dextrose gel.

### Future directions

Clinical research in low resource settings is difficult. Yet current evidence from the developed world indicates dextrose gel could be useful in low-resource settings, and its use is in line with best practice guidelines [42, 45, 48]. Therefore, we advocate the urgent need for robust clinical trials in low-resource settings to investigate current clinical practices, and determine the efficacy, viability, and cultural appropriateness of dextrose gel, both as a prophylaxis and treatment to improve outcomes for babies and families [74, 76]. Importantly, well designed follow-up studies of children who receive dextrose gel as newborns to determine long-term neurodevelopmental outcomes are also essential.

## Conclusion

It has been 25 years since the WHO identified the prevention, screening and management of neonatal hypoglycaemia as a global priority [1] and yet there are still major barriers to effective care that have not been addressed. Research in LLMIC is limited, but there are clear practice changes that could reduce the incidences and burden of neonatal hypoglycaemia, including increased screening, improved methods of glucose screening, evidence based and culturally appropriate protocols for the treatment and screening of at-risk babies, improved staff and parental education and the use of dextrose gel. However, the first step to strengthening the health services and improving neonatal health outcomes in low-resource settings is to conduct co-designed clinical trials in these countries to inform culturally sensitive evidenced-based practice and reduce the inequities that babies and families in low resource settings are currently facing.
